# Epidermal PPARγ influences subcutaneous tumor growth and acts through TNF-α to regulate contact hypersensitivity and the acute photoresponse

**DOI:** 10.18632/oncotarget.21002

**Published:** 2017-09-18

**Authors:** Raymond L. Konger, Ethel Derr-Yellin, Jeffrey B. Travers, Jesus A. Ocana, Ravi P. Sahu

**Affiliations:** ^1^ Department of Pathology & Laboratory Medicine, Indiana University School of Medicine, Indianapolis, IN, USA; ^2^ Department of Dermatology, Indiana University School of Medicine, Indianapolis, IN, USA; ^3^ Department of Pharmacology & Toxicology, Wright State University, Dayton, OH, USA

**Keywords:** contact hypersensitivity, immunosuppression, peroxisome proliferator-activated receptor Γ, ultraviolet, tumor necrosis factor alpha

## Abstract

It is known that ultraviolet B (UVB) induces PPARγ ligand formation while loss of murine epidermal PPARγ (*Pparg*-/-^epi^) promotes UVB-induced apoptosis, inflammation, and carcinogenesis. PPARγ is known to suppress tumor necrosis factor-α (TNF-α) production. TNF-α is also known to promote UVB-induced inflammation, apoptosis, and immunosuppression. We show that *Pparg*-/-^epi^ mice exhibit increased baseline TNF-α expression. Neutralizing Abs to TNF-α block the increased photo-inflammation and photo-toxicity that is observed in *Pparg-/-*^epi^ mouse skin. Interestingly, the increase in UVB-induced apoptosis in *Pparg-/-*^epi^ mice is not accompanied by a change in cyclobutane pyrimidine dimer clearance or in mutation burden. This suggests that loss of epidermal PPARγ does not result in a significant alteration in DNA repair capacity. However, loss of epidermal PPARγ results in marked immunosuppression using a contact hypersensitivity (CHS) model. This impaired CHS response was significantly alleviated using neutralizing TNF-α antibodies or loss of germline *Tnf*. In addition, the PPARγ agonist rosiglitazone reversed UVB-induced systemic immunosuppression (UV-IS) as well as UV-induced growth of B16F10 melanoma tumor cells in syngeneic mice. Finally, increased B16F10 tumor growth was observed when injected subcutaneously into *Pparg*-/-^epi^ mice. Thus, we provide novel evidence that epidermal PPARγ is important for cutaneous immune function and the acute photoresponse.

## INTRODUCTION

Peroxisome proliferator-activated receptors (PPAR) are ligand-activated nuclear transcription factors which exhibit binding affinity for a diverse range of bioactive lipid and xenobiotic agents [1]. Three different PPAR isotypes have been described (PPARα, PPARβ/δ, and PPARγ), each of which forms a heterodimer with the retinoid X-receptor (RXR). These heterodimeric complexes can then activate the transcription of target genes by binding to peroxisome proliferator response elements (PPRE). PPARs are also known to suppress the transcription of selected genes, particularly pro-inflammatory gene products, through poorly understood ligand-dependent and ligand-independent transrepressive mechanisms (reviewed in [2]). The three PPARs exhibit different ligand specificities, tissue expression patterns and transcriptional targets, although all three isotypes are expressed in murine and human keratinocytes [1]. PPARγ is best known for its role in adipogenesis and insulin sensitization. This has led to the use of synthetic PPARγ agonists (pioglitazone and rosiglitazone) as anti-diabetic agents [1]. PPARγ ligands have also been shown to have anti-cancer activity in tumor cell lines, in murine tumor models and in human clinical or epidemiological studies [3, 4].

Ultraviolet (UV) exposure from sunlight can be divided into UVA (320–400 nm), UVB (290–320 nm), and UVC (200-290 nm) [1]. However, UVC is filtered by the atmosphere and does not play a significant role in photobiology. Cutaneous carcinogenesis is primarily mediated by UVB exposure. We have shown that mice lacking epidermal PPARγ *(Pparg*-/-^epi^ mice) are prone to increased skin tumor incidence and multiplicity following multiple UVB treatments [5]. In addition, mice lacking epidermal PPARγ or its heterodimerization partner (RXRα) and mice with germ-line deletion of one PPARγ allele exhibit enhanced chemical carcinogenesis [6, 7]. In addition to increased tumor formation, *Pparg*-/-^epi^ mice exhibited increased sensitivity for photoinflammation, phototoxicity, epidermal hyperplasia and an increase in p53+ epidermal cells following chronic UVB treatments [5].

UVB exposure is immunosuppressive, acting to suppress Th-1 mediated delayed type hypersensitivity and contact hypersensitivity responses [8]. Studies in humans and mice indicate that UV-induced immunosuppression (UV-IS) plays an important role in photocarcinogenesis and anti-tumor immune responses [8, 9]. The ability of UV-IS to suppress anti-tumor immune responses is seen by the ability of UVB treatment to promote B16F10 tumor growth in C57BL/6 mice [9].

Various lines of evidence suggest that PPARγ may act to alter immune function. PPARγ expressed in various immune cells plays a role in modifying the immune function of these cells [10]. In addition, systemic PPARγ ligand treatment has been shown to inhibit allergic responses in the skin of mice and humans [11, 12]. Other studies suggest that PPARγ activation in non-immune cells may also regulate immune function through paracrine signaling. Transgenic mice expressing a dominant negative PPARγ in type II alveolar cells exhibited increased production of inflammatory mediators TNF-α, interleukin (IL)-1, and IL-6 that were necessary for an increase in immature myeloid cells and decreased T-cells within the lungs [13]. While it is unclear whether this led to an immunocompromised state *in vivo*, isolated immature pulmonary myeloid cells had the capacity to suppress T-cell activation *in vitro* [13].

DNA readily absorbs UVB, and this absorption can directly induce DNA lesions (cyclobutane pyrimidine dimer (CPD) or 6, 4-photoproduct formation) that can lead to DNA mutations [14]. That *Pparg*-/-^epi^ mice exhibit a pronounced increase in sensitivity to UVB-induced apoptosis suggests a potential role for epidermal PPARγ in regulating UVB-induced mutation burden [5]. PPARs have been shown to regulate the expression of DNA damage response genes and oxidative stress [15, 16]. However, the ability of PPARs to regulate DNA repair and mutational events is largely unexplored.

Tumor necrosis factor alpha (TNF-α) is a primary inflammatory cytokine that is induced in keratinocytes by UV exposure and plays a key role in promoting photoinflammation, phototoxicity and UV-IS [17, 18]. Mice lacking either TNF-α or TNF receptors are also resistant to cutaneous chemical carcinogenesis [19, 20]. The ability of TNF-α to promote immune suppression has been attributed to multiple mechanisms, including depressed Langerhans cell migration and promotion of either myeloid-derived suppressor cell or mast cell accumulation [18, 21, 22]. TNF-α is initially produced as a 26 kDa transmembrane protein (tmTNF-α) that is cleaved to produce the 17 kDa soluble TNF-α (solTNF-α) [23]. Both tmTNF-α & solTNF-α have biological activity, with tmTNF-α acting as both a ligand and a receptor [24]. Importantly, while solTNF-α acts to promote inflammatory events, tmTNF-α is proposed to act to trigger resolution of the inflammatory response [25].

Given the well-known ability of PPARγ activation to suppress TNF-α expression in other cell types, as well as the known ability of TNF-α to mediate UVB-induced acute inflammation, apoptosis,and carcinogenesis, we examined whether the acute UVB responses that we have previously seen in *Pparg*-/-^epi^ mice could be attributable to downstream TNF-α signaling. Given that cancer is ultimately dependent on mutational events, we also examined whether loss of epidermal *Pparg* altered UVB-induced DNA damage and mutation frequency. Finally, we also sought to determine whether epidermal PPARγ acts to mediate cutaneous immune responses by examining both CHS and anti-tumor immune responses.

## RESULTS

### *Pparg-/-^epi^* mice exhibit an increase in TNF-α production

**Figure 1:**
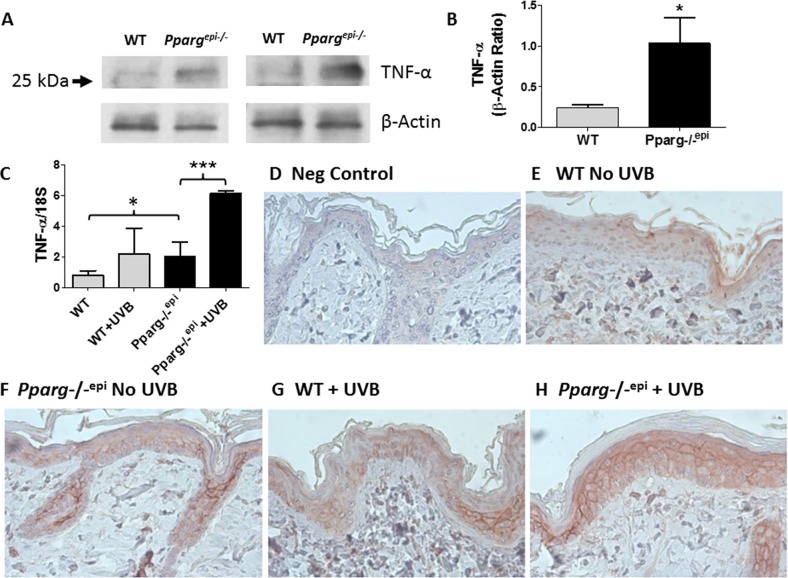
Increased TNF-α consistent with tmTNF-α is observed in *Pparg*-/-^epi^ mice both prior to and after UVB irradiation **(A)** Increased TNF-α protein is produced under normal physiologic conditions in the epidermis of *Pparg*-/-^epi^ mice relative to WT mice. WT and *Pparg*-/-^epi^ mice were euthanized and the epidermis was removed from the dorsal skin for protein extraction and TNF-α immunoblot. After stripping, a β-actin immunoblot was performed to assess loading. Representative immunoblots from epidermal preparations of two different WT and *Pparg*-/-^epi^ mice are shown. Only an approximately 26 kDa protein was observed by anti-TNF-α immunoblot. A 17 kDa protein that would correspond to solTNF-α was not observed (not shown). **(B)** Quantitative assessment of TNF-α protein in epidermal lysates normalized to β-actin. TNF-α protein levels were normalized to β-actin. Mean & SEM, ^*^, p<0.05; n= epidermal lysates from 5 mice/genotype. **(C)** TNF-α mRNA expression in WT and *Pparg*-/-^epi^ mice. Mice were irradiated with 1500 J/m^2^ UVB. After 24 hours, the mice were euthanized, the skin excised, frozen in liquid N_2_, and the epidermis was scraped off for RNA extraction & quantitative RT-PCR (qRT-PCR). TNF-α expression in epidermal scrapings was normalized to 18 S rRNA. Mean and SEM, n=4-6 mice per experimental group. ^*^, p<0.05; ^***^, p<0.001, 2-tailed t-test. **(D-H)** TNF-α protein expression localized to the epidermis following UVB irradiation is increased in *Pparg*-/-^epi^ mice. Mouse skin was excised 24 hours after a 1500 J/m^2^ UVB treatment and formalin fixed and paraffin-embedded. Immunolabeling was then performed using an anti-TNF-α antibody (α-TNF). D.) Representative photomicrograph of a control in which staining was performed in the absence of the primary antibody. E-H.) Representative images of TNF-α expression within the epidermis of WT and *Pparg*-/-^epi^ mice treated with or without UVB. Both UVB treatment and loss of epidermal *Pparg* were associated with an increase in staining consistent with localization to the cell membrane. All images are 400x and are representative of TNF-α immunolabeling performed on at least 3 different mice per treatment group.

Given that PPARγ activation is known to suppress TNF-α production, we measured epidermal TNF-α expression in wildtype (WT) and *Pparg*-/-^epi^ mice. We verified that TNF-α protein was increased by approximately 4-fold in protein lysates prepared from the epidermis of *Pparg*-/-^epi^ mice (Figure 1A&1B). The size of the TNF-α protein seen by immunoblot is consistent with the full-length 26 kDa tmTNF-α. While our antibody reportedly detects the 17 kDa solTNF-α cleavage product, we did not observe a 17 kDa immunoreactive band consistent with solTNF-α in epidermal protein lysates (not shown).

UVB treatment is known to induce TNF-α production in the epidermis [17, 19]. We therefore examined how loss of epidermal *Pparg* alters TNF-α expression following an acute UVB exposure. In Figure 1C, we show that untreated *Pparg-/-^epi^* mice express significantly higher levels of TNF-α transcripts relative to WT mice. A synergistic effect on TNF-α expression was noted when *Pparg-/-^epi^* mice were treated with UVB relative to UVB-treated WT mice. Finally, we examined TNF-α protein localization by immunohistochemistry (Figures 1D-1H). Epidermal TNF-α protein expression was observed in all mice, but was increased in non-UVB treated *Pparg*-/-^epi^ epidermis relative to non-UVB treated WT mice. As with the RT-PCR studies, UVB-treated WT mice and non-UVB-treated *Pparg*-/-^epi^ mice showed similar levels of TNF-α expression, while *Pparg*-/-^epi^ mice treated with UVB showed the highest level of epidermal expression. Finally, consistent with our immunoblot data, UVB-treated WT mouse epidermis and basal or UVB-treated *Pparg*-/-^epi^ mouse epidermis exhibits a characteristic cell membrane localization pattern that is consistent with a transmembrane form of TNF-α (Figures 1F-1H).

### The enhanced acute edematogenic response to a single UVB exposure that is seen in *Pparg*-/-^epi^ mice is blocked by neutralizing anti-TNF-α antibodies

**Figure 2:**
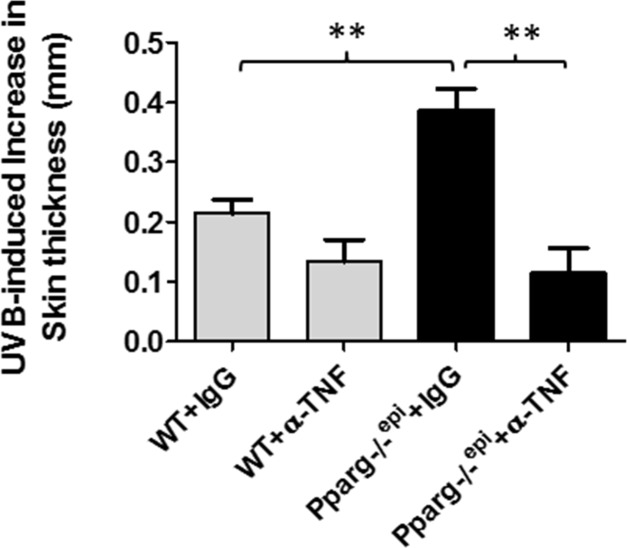
Neutralizing antibodies to TNF-α block the augmented increase in skin thickness that occurs following an acute UVB exposure of *Pparg*-/-^epi^ mice WT and *Pparg*-/-^epi^ mice were injected intraperitoneally with 250 μg of anti-TNF-α antibodies (α-TNF) or an isotype control antibody (IgG). One day later, anesthetized mice were treated with or without 1500 J/m^2^ UVB. After 24 hours, the mice were euthanized, the dorsal UVB-treated skin removed and snap frozen in liquid N_2_ for skin thickness measurements. Data is shown as the mean and SEM of the UVB-induced increase in skin thickness after subtracting non-UVB treated skin ^**^, *p*<0.01, 2-tailed t-test. n=4-5 mice/group.

UVB-induced inflammation in mouse skin is initially assessed by the cutaneous edema response, which can be measured as a change in skin thickness at 24 hours post-UVB treatment [26]. In Figure 2, we show that *Pparg-/-^epi^* mice exhibit a pronounced increase in UV-induced skin thickness 24 hours after UVB irradiation [5]. Since TNF-α is a well-known pro-inflammatory mediator, we also examined whether increased TNF-α expression in *Pparg-/-^epi^* mice was involved in the increased sensitivity of *Pparg-/-^epi^* mice to photo-inflammation. In Figure 2, we also show that anti-TNF-α antibody treatment blocked the augmented UVB-induced inflammatory response in *Pparg-/-^epi^* mice.

### The augmented apoptotic response to a single UVB exposure that is observed in *Pparg*-/-^epi^ mice is also blocked by anti-TNF-α treatment

**Figure 3:**
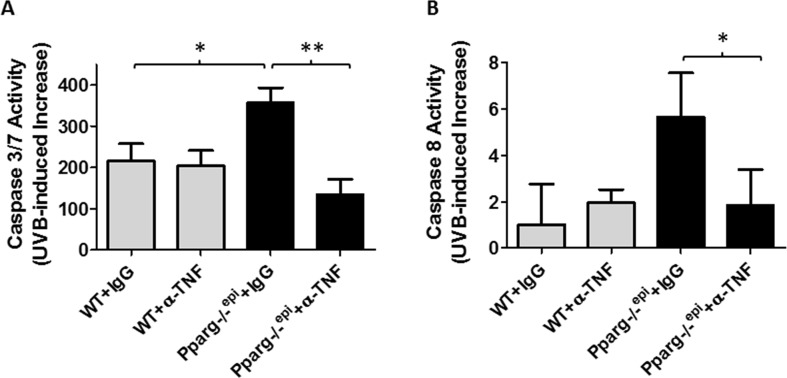
Neutralizing antibodies to TNF-α block the augmented increase in UVB-induced apoptosis in *Pparg*-/-^epi^ mice WT and *Pparg*-/-^epi^ mice were treated with IgG or anti-TNF-α antibodies, then treated with or without UVB as described in Figure 2. After 24 hours, the mice were euthanized and treated skin was snap frozen in liquid N_2_. Caspase 3/7 or caspase 8 activity was then assessed in epidermal scrapings. **(A)** Caspase 3/7 activity is shown for the UVB-treated animal groups after subtracting background activity from the relevant non-UVB treated groups. Results represent the mean and SEM from n=6, n=5, n=10, or n=5 mice for the WT+IgG, WT+α-TNF, *Pparg*-/-^epi^+IgG, and *Pparg*-/-^epi^+α-TNF groups, respectively. **(B)** Caspase 8 activity is shown for the UVB-treated animal groups after subtracting background activity from the relevant non-UVB treated groups. Results represent the mean and SEM from n=3, n=3, n=4, or n=4 mice for the WT+IgG, WT+α-TNF, *Pparg*-/-^epi^+IgG, and *Pparg*-/-^epi^+α-TNF groups, respectively. ^*^, p<0.05; ^**^, p<0.01, 2-tailed t-test.

TNF-α augments UVB-induced apoptosis in keratinocytes [27]. We therefore examined whether anti-TNF-α neutralizing antibodies would suppress the augmented apoptosis that we have observed in *Pparg-/-^epi^* mice 24 hours after UVB treatment [5]. As expected, we show that *Pparg-/-^epi^* mice treated with control IgG exhibit higher caspase 3/7 activities after UVB treatment relative to WT mice (Figure 3A). Caspases 3 and 7 act downstream of both the intrinsic and extrinsic apoptotic pathways, while TNF-α can induce apoptosis through binding to the TNF receptor 1, thus activating caspase 8 in the extrinsic apoptosis pathway [28]. In Figure 3B, we show that UVB treatment induces an augmented activation of caspase 8 in *Pparg-/-^epi^* mice, and that this activation is blocked by anti-TNF-α antibodies.

### *Pparg-/-^epi^* mice fail to exhibit alterations in UV-induced CPD repair or mutation frequency or proliferative responses to UVB

**Figure 4:**
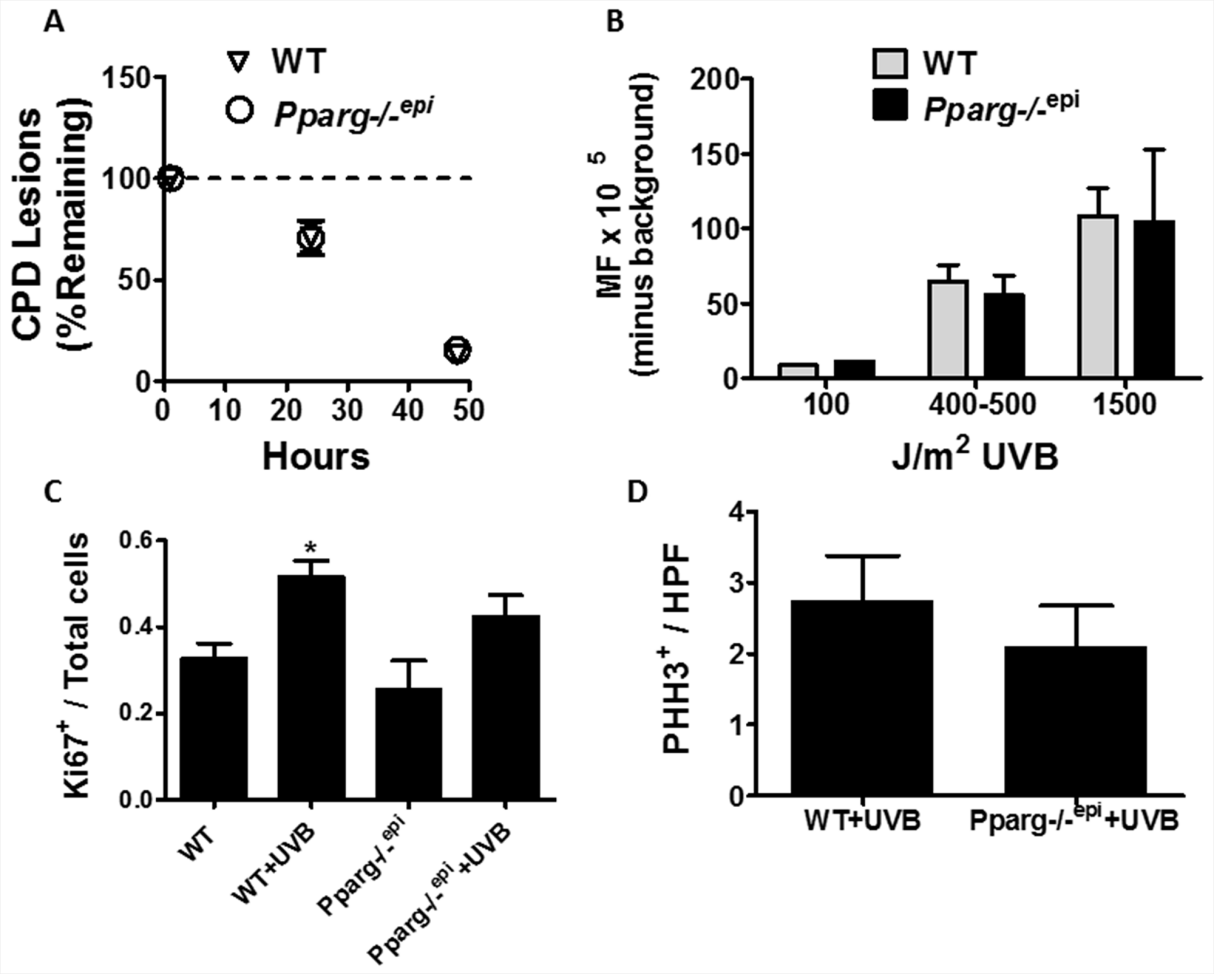
Cyclobutane pyrimidine dimer (CPD) repair rates, mutation frequency, and epidermal hyperplasia are not altered in *Pparg-/-^epi^* mice following a single UV treatment **(A)** *UVB-induced CPD clearance is not altered in Pparg-/-^epi^ mice*. CPD clearance rates are shown for WT and *Pparg-/-^epi^* mice at 1, 24, and 48 hours after the treatment with a single 1500 J/m^2^ dose of UVB. Results are shown as the mean and SEM of the % of CPD lesions remaining relative to the 1 hour time point (n=5-7 mice per experimental group and time point). **(B)** *UVB-induced mutation frequency (MF) is not altered by epidermal PPARγ status*. Big Blue^®^ mice backcrossed into the SKH-1 background were treated with 0, 100, 400-500 or 1500 J/m^2^ UVB. The mice were then euthanized 5 days later and the skin removed. Epidermal DNA was then utilized for lambda phage insertion and MF detection by plaque formation in a lawn of *E. coli*. After subtracting the average background (no UVB) MF, the mean and SEM of the MF (mutant plaques/total plaques x 10^5^) is shown. The results represent data from n=1 mouse (100 J/m^2^), n=3 mice (400-500 J/m^2^) or n=4-5 mice (1500 J/m^2^) for each genotype. C&D.) *Loss of epidermal Pparg does not promote epidermal hyperplasia following a single UVB treatment*. WT and *Pparg*-/-^epi^ mice were treated with or without a single dose of 1500 J/m^2^ UVB. At 72 hours, the mice were euthanized, the UVB-treated skin and non-UVB treated control skin was excised and Ki67 immunolabeling (C) or phospho-histone H3 (PHH3) immunolabeling (D) was performed. For Ki67 immunolabeling, the immunopositive cells are shown as a ratio of total epidermal cells. Mean and SEM, n=3-4 mice / group. For PHH3 immunolabeling, the results represent the number of PHH3+ epidermal cells per high power (400x) field (mean and SEM, n=4 mice / group).

**Figure 5:**
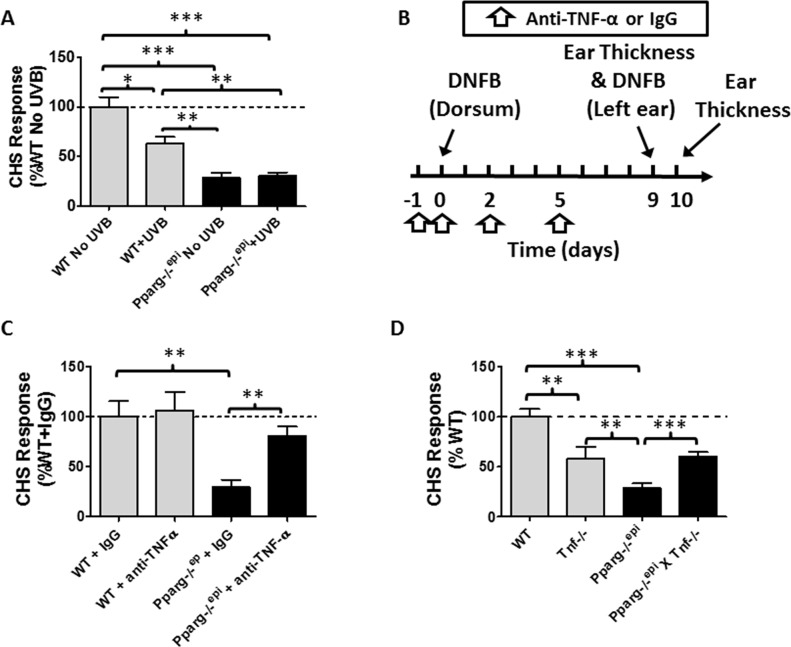
*Pparg-/-^epi^* mice exhibit a marked defect in the contact hypersensitivity (CHS) response that is not affected by UVB exposure but is suppressed by anti-TNF-α neutralizing antibodies **(A)** *Pparg-/-^epi^* mice are immunosuppressed using a CHS model. *WT* and *Pparg*-/-^epi^ mice were treated with or without UVB (7500 J/m^2^) on the dorsal skin at a site distant to where the mice were initially sensitized to the hapten dinitrofluorobenzene (DNFB). Initial DNFB sensitization of the mice occurred five days after UVB treatment. Nine days after the initial DNFB sensitization, ear thickness was measured and DNFB was applied to one ear while the other ear was treated with vehicle. The change in ear thickness was assessed 24 hours after DNFB challenge and is plotted as the change in ear thickness after subtracting the thickness of the contralateral ear treated with vehicle alone. Results represent the change in ear thickness measurements as a percent of non-UVB treated control SKH1 wildtype mice. Results represent the mean ± SEM for 6 non-UVB treated and 4 UVB-treated mice per genotype. **(B)** *Timeline for neutralizing anti-TNF-a studies*. WT SKH1 and *Pparg*-/-^epi^ mice were injected intraperitoneally (ip) with 250 μg of either anti-TNF-α or isotype control on days -1, 0, 2 and 5. On day 0, the dorsal skin of the mice were sensitized with 0.5% DNFB. CHS responses to DNFB challenge were assessed 9 days later by measuring ear thickness and applying DNFB to the left ear and vehicle to the right ear. Ear thickness was re-measured 1 day later. **(C)** *Anti-TNF-α antibody treatment (anti-TNF-α) reversed the impaired CHS response observed in Pparg-/-^epi^ mice, but has no effect on CHS responses in WT mice*. Mice were treated as described in Figure 5C. Results represent the percent change in ear thickness measurements compared to wildtype SKH1 mice treated with control IgG alone. Mean and SEM for n=5-6 mice per experimental group. (D) *Germline loss of Tnf (Tnf-/-) partially reverses the impaired CHS response in Pparg-/-^epi^ mice*. WT mice, *Tnf-/-* mice, *Pparg*-/-^epi^ mice, and *Pparg*-/-^epi^ x *Tnf-*/- double knockout mice were sensitized with DNFB. After 7 days, a DNFB challenge was applied to the left ear and ear thickness was measured. CHS responses (as a percentage of WT response) were assessed. Mean and SEM, n=8 (WT), n=16 (*Pparg*-/-^epi^), n=6 (*Tnf-/-*), and n=10 (*Pparg*-/-^epi^ X *Tnf-/-*). ^*^, p<0.05; ^**^, *p*<0.01; ^***^, *p*<0.001, 2-tailed *t*-test. In the absence of any stimulation, there was no significant difference in baseline ear thickness measurements between WT, *Pparg*-/-^epi^, *Tnf*-/-, or *Pparg*-/-^epi^ X *Tnf*-/- mice (0.247 ± 0.019 mm, n=18; vs 0.251 ± 0.024 mm, n=27 vs. 0.235 ± 0.014, n=10 vs 0.249 ± 0.012, n=14; respectively).

Mice lacking epidermal PPARγ or its heterodimeric partner RXRα exhibit both enhanced cutanaeous carcinogenesis and an increase in UV-induced apoptosis (Figure 3 and [5, 29]). An increase in apoptosis might be indicative of increased DNA damage or an impaired DNA damage response. We therefore examined whether loss of epidermal PPARγ promoted the formation of UVB-induced DNA damage or mutation frequency. UVB-induced mutations are largely thought to be mediated by the formation of CPD lesions in the DNA, which are cleared by the nucleotide excision repair (NER) pathway [14]. Using an EIA-based assay, we failed to observe any change in the rate of CPD clearance in *Pparg-/-^epi^* mice relative to WT mice (Figure 4A)(Supplementary Figure 1A demonstrates the linearity of the EIA assay). We also failed to observe a significant difference in initial CPD lesions between WT and *Pparg*-/-^epi^ mice (Supplementary Figure 1B). This strongly suggests that the NER process is not altered in *Pparg-/-^epi^* mice.

While clearance of CPD lesions is intact in *Pparg*-/-^epi^ mice, increased mutations can occur with normal CPD clearance, such as in individuals with Xeroderma pigmentosa variant (XPV) [30]. In addition, RXRα mice were shown to exhibit an increase in oxidative DNA damage following UV treatment, particularly in melanocytes [29]. Oxidized DNA lesions are not cleared by NER, but are repaired by base excision repair [31]. It is therefore possible that UVB-induced oxidative DNA damage could account for an increase in mutational events independent of CPD lesions. We therefore examined whether *Pparg-/-^epi^* mice exhibited a change in mutation frequency following an acute UVB treatment. To perform this analysis, we utilized *Pparg-/-^epi^* mice that were crossed with Big Blue^®^ mice containing the λLIZ shuttle vector for mutation detection [32]. Using WT Big Blue mice and *Pparg*-/-^epi^ Big Blue mice, we failed to find a significant difference in mutation frequency (MF) following UVB treatment (Figure 4B). The above data strongly suggests that the increased photocarcinogenesis that we have previously observed in *Pparg*-/-^epi^ mice is not due to an increase in susceptibility to UVB-induced initiating mutational events.

Finally, an increase in epidermal proliferation was noted in non-tumor containing areas of *Pparg*-/-^epi^ mice relative to wildtype mice that had been treated chronically for 24 weeks with UVB [5]. This increase in epidermal hyperplasia following multiple UVB treatments could be due to the ability of PPARγ activation to directly suppress keratinocyte growth. If so, we reasoned that loss of epidermal PPARγ should result in an increase in the epidermal hyperplasia that is seen following a single UVB treatment [26]. In mice, epidermal hyperplasia peaks at approximately 72 hours post-UVB treatment [26]. In Figure 4C, the expression of the proliferation marker Ki67 within the epidermis of wildtype (WT) and *Pparg*-/-^epi^ mice at baseline and 72 hours after UVB treatment is shown. There was no appreciable difference in epidermal Ki67+ cells in WT or *Pparg*-/-^epi^ mice in the absence of UVB. While UVB induced an increase in Ki67 immunolabeling at 72 hours in both WT and *Pparg*-/-^epi^ mice, the presence or absence of *Pparg* did not significantly affect the results. To verify that epidermal PPARγ status fails to impact the UVB-induced mitogenic effect, we utilized a second marker of epidermal proliferation (phospho-histone H3) (Figure 4D). Again, no significant difference was noted in the UVB-induced proliferative response from WT or *Pparg*-/-^epi^ mice. We have also failed to demonstrate that PPARγ ligands alter the proliferation of primary human keratinocytes or mouse squamous cell carcinoma cells *in vitro* (data not shown). Thus, epidermal PPARγ does not likely play a significant direct role in mouse keratinocyte proliferative responses, either under basal conditions or following an acute UVB exposure.

### Contact hypersensitivity is impaired in *Pparg-/-^epi^*mice

UVB-induced TNF-α production has been shown to promote UV-IS [18]. We therefore examined whether CHS responses were altered in *Pparg-/-^epi^* mice. In Figure 5A, we show that *Pparg-/-^epi^* mice exhibit an approximately 70% reduction in the CHS response to the contact allergen, 2,4-dinitrofluorobenzene (DNFB). This CHS suppression was significantly greater than that observed for UVB treatment of WT SKH1 mice. The addition of UVB treatment to *Pparg-/-^epi^* mice resulted in no additional change to the impaired CHS response observed in non-UVB treated *Pparg-/-^epi^* mice.

To determine whether the up-regulated TNF-α production observed in *Pparg-/-^epi^* mice could be playing a role in the impaired CHS response observed in these mice, we treated WT and *Pparg*-/-^epi^mice with neutralizing anti-TNF-α antibodies and again assessed CHS responses using the strategy outlined in Figure 5B. In Figure 5C, we show that anti-TNF-α treatment had no significant effect on the CHS response in WT mice, but significantly reversed the immunosuppression seen in *Pparg*-/-^epi^ mice. Finally, we determined whether loss of germline *Tnf* would reverse the impaired CHS response in *Pparg*-/-^epi^ mice. We therefore generated *Pparg*-/-^epi^ x *Tnf*-/- crosses. In Figure 5D, we show that loss of *Tnf* alone resulted in a reduction in the CHS response. This reduction in CHS responses was not as pronounced as that observed in *Pparg*-/-^epi^ mice. Importantly, the combined loss of *Tnf* in *Pparg*-/-^epi^ mice reversed the impaired CHS response to that observed with loss of *Tnf* alone.

### Rosiglitazone treatment blocks systemic UVB-induced immunosuppression (UV-IS) and UVB-induced B16F10 tumor growth

**Figure 6:**
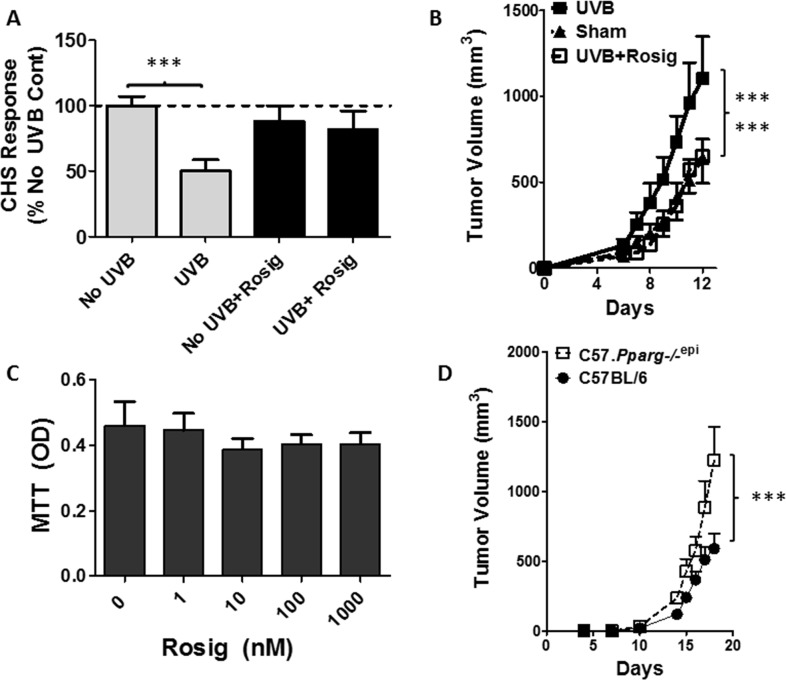
Rosiglitazone (Rosig) reverses UVB-induced immunosuppression and UVB-induced B16F10 tumor growth while intradermal B16F10 tumor growth is increased in mice lacking epidermal *Pparg* **(A)** *Rosig treatment reverses UVB-induced immunosuppression (UV-IS)*. SKH1 mice were treated with Rosig or normal water for 10 days prior to treatment of the shaved dorsal epidermis with/without 7500 J/m^2^ UVB to a site distant from DNFB sensitization. Rosig treatment was maintained throughout the experiment. Following DNFB sensitization, CHS responses to DNFB reapplication to non-UVB treated ears were measured 9 days later as in Figure 5. Results are shown as a percentage change from non-UVB/non-Rosig treated control mice (No UVB) and are depicted as the mean and SEM (n=7 mice / group). ^***^, *p*<0.001; 2-tailed t-test. (B) *Rosig treatment reverses UVB-induced B16F10 tumor growth*. C57BL6 mice were introduced to Rosig or water for 10 days prior to tumor cell injection. Just prior to tumor cell injection, and on days 6 and 12 thereafter, anesthetized mice were treated with/without 5000 J/m^2^ UVB at a site distant from tumor cell injection. B16F10 melanoma tumor cells (5×10^5^) were injected subcutaneously into the rear hindlimbs and tumor growth monitored. Mean and SEM shown (n=12/group). Tumor growth was significantly increased in UVB treated mice relative to non-UVB treated (Sham) or UVB treated mice that were also treated with Rosig (UVB+Rosig). ^***^, p<0.001; 2-way ANOVA. **(C)** Rosig does not significantly alter B16F10 tumor cell growth *in vitro*. B16F10 tumor cells were grown in the presence of vehicle (0) or increasing concentrations of Rosig. After 48 hours, cell proliferation was assessed by quantitating viable cells by MTT assay. The results represent the mean and SEM of n=5 separate assays done in quadruplicate wells. **(D)** *Subcutaneous B16F10 tumors grow significantly faster in syngeneic mice lacking epidermal Pparg (C57.Pparg-/-^epi^ mice)*. C57BL6 mice with epidermal specific deletion of *Pparg* were generated (C57. *Pparg*-/-^epi^ mice) to allow for syngeneic tumor growth studies. B16F10 tumor cells (5×10^4^) were injected into the hindlimbs of WT or C57.*Pparg*-/-^epi^ mice and tumor size was monitored. At day 18, the mean tumor size in C57.*Pparg*-/-^epi^ mice was 2.07-fold greater than that observed in WT mice (mean ± SEM of 590.9 ± 334 and 1221.9 ± 677.6 for 10 tumors in WT and 8 tumors in C57*.Pparg*-/-^epi^ mice respectively). ^***^, *p*<0.001, 2-way ANOVA,.

We next examined whether the PPARγ agonist rosiglitazone (Rosig) could reverse UV-IS. Control mice treated with a high dose of UVB at a site distant from the site of DNFB sensitization exhibited a reduction in ear inflammation following subsequent DNFB challenge (Figure 6A). Rosiglitazone treatment alone had no effect on the CHS response in non-UVB treated mice. However, rosiglitazone blocked the ability of UVB treatment to suppress the CHS response.

Following the transplant of B16F10 melanoma tumor cells into syngeneic mice, we have demonstrated that UV-IS promotes the growth of the transplanted tumors [9]. In Figure 6B, we show that rosiglitazone treatment blocks the ability of UVB to promote B16F10 tumor growth. Since B16F10 cells express PPARγ [33], it is possible that rosiglitazone treatment suppressed B16F10 tumor growth through direct effects on the tumor cells. We therefore determined whether rosiglitazone affected B16F10 tumor cell growth *in vitro*. We did not observe a significant decrease in B16F10 tumor cell growth at concentrations up to 1 μm, well above the binding affinity for rosiglitazone for PPARγ (EC_50_ = 23 nM) [34] (Figure 6C). We also examined whether the growth of subcutaneous B16F10 tumors were altered in *Pparg*-/-^epi^ mice derived in the C57BL/6 background (C57.*Pparg*-/-^epi^ mice). We observed a nearly 2-fold increase in tumor growth in C57.*Pparg*-/-^epi^ mice relative to tumors transplanted into wildtype C57BL/6 mice.

## DISCUSSION

We have previously shown that mice lacking epidermal PPARγ are highly susceptible to UVB-induced photoinflammation, phototoxicity and photocarcinogenesis [5]. The two major findings from this current study are that TNF-α plays a key role in the ability of epidermal PPARγ to alter UVB-induced acute inflammation and apoptosis and that loss of epidermal PPARγ also results in suppression of normal contact hypersensitivity responses while the PPARγ agonist rosiglitazone reverses UVB-induced immune suppression. We also provide evidence that PPARγ-dependent suppression of basal TNF-α production serves as a major mechanism for this observed immunosuppressive activity. Thus, we propose that PPARγ-dependent suppression of TNF-α signaling plays a key mechanistic role in the acute photo-response and cutaneous immune function. The potential importance of our findings to cutaneous neoplasia is illustrated by the ability of the PPARγ ligand rosiglitazone to reverse both UVB-induced impairment in CHS responses as well as UVB-induced B16F10 tumor growth. Finally, we show that B16F10 melanoma tumor growth is enhanced in syngeneic C57.*Pparg*-/-^epi^ mice, indicating that loss of epidermal PPARγ acts through indirect mechanisms to regulate tumor growth.

PPARγ activation is known to suppress inflammatory signaling and TNF-α production in other cell types [2, 10, 13]. TNF-α production is known to mediate UVB-induced inflammatory responses [17, 18]. We now show that TNF-α expression is upregulated in *Pparg*-/-^epi^ mice and that TNF-α neutralizing antibodies suppress the augmented photo-inflammatory response that is observed in *Pparg*-/-^epi^ mice. We also found that increased epidermal TNF-α expression is observed in *Pparg*-/-^epi^ mice under basal conditions (no UVB). This basal increase presumably represents a sustained, rather than transient, elevation of TNF-α. While no specific stimuli was introduced, basal TNF-α expression likely occurs in response to everyday cutaneous stressors (e.g. sloughing of skin due to contact with other mice and the cage environment or exposure to commensal flora). In addition, we provide evidence that the transmembrane form of TNF-α (tmTNF-α) is upregulated in the epidermis of *Pparg*-/-^epi^ mice. Since *Pparg*-/-^epi^ mice exhibit increased photocarcinogenesis, a potential role for tmTNF-α as a downstream mediator of epidermal PPARγ is interesting given that studies using *Tnf*, *Tnfr1* or *Tnfr2* knockout mice or mice treated with anti-TNF-α antibodies suggest that TNF-α acts to promote cutaneous carcinogenesis in mice [19, 20]. However, there has been an inconsistent and controversial correlation between anti-TNF-α therapies and increased risk for non-melanoma skin cancer in humans [35–37]. While neutralizing anti-TNF-α antibodies were thought to act primarily by blocking solTNF-α activity, more recent evidence suggests that some effects that are elicited by these agents may occur through interaction with tmTNF-α [24]. It is therefore possible that the ability of these therapies to suppress or promote carcinogenesis may be related to the relative ability of specific anti-TNF-α agents to block tmTNF-α versus solTNF-α signaling.

Increased apoptosis is commonly thought to primarily protect against mutational events and subsequent tumorigenesis. The heightened sensitivity of *Pparg-/-*^epi^ mice to increased phototoxicity therefore suggests a possible role for epidermal PPARγ in regulating UVB-induced DNA damage or repair. UVB-induced DNA damage within the epidermis is also thought to initiate the signaling cascade that leads to local UV-IS [38]. However, the inverse correlation between apoptosis and mutational events is context dependent: This protection is dependent on appropriate coordination between cell cycle arrest, apoptotic signaling and DNA repair processes [39, 40]. Our data indicates that cyclobutane pyrimidine dimer (CPD) mutation clearance and overall mutation frequency are not altered in *Pparg*-/-^epi^ mice. It therefore seems unlikely that PPARγ plays a key role in regulating DNA repair in keratinocytes. Our conclusion is supported by a study showing that mice lacking epidermal RXRα, which partners with PPARγ to regulate PPARγ-dependent transcriptional activity, exhibit increased UVB-induced apoptosis coupled to normal CPD clearance [29]. A limitation of our studies was that we only examined the effects of a single UVB exposure on CPD clearance and mutation frequency. Given the marked increase in photocarcinogenesis observed in *Pparg*-/-^epi^ mice, we cannot exclude the possibility that loss of keratinocyte PPARγ plays a role in the long-term adaptive response of the skin to repetitive UVB exposures. In addition, given the increased apoptosis in the setting of normal DNA damage repair and mutation frequency, it seems likely that PPARγ plays a role in apoptotic signaling that is downstream of the initial DNA damage repair process. It is possible that this is mediated by increased TNF-α, as TNF-α synergizes with UVB to induce apoptosis in keratinocytes [27].

Various studies have shown that activation of PPARγ within various cells of the immune system can alter their function [41–44]. Additional pharmacologic studies have suggested a role for PPARγ in regulating adaptive immune function [45]. However, these studies do not rule out off-target pharmacologic effects [45]. Our studies add to these previous results by conclusively demonstrating that PPARγ expression within epithelial cells can indirectly regulate immune function using the well-established *in vivo* contact hypersensitivity (CHS) assay of T-cell mediated immunity. We observed an approximately 70% reduction in CHS responses in *Pparg*-/-^epi^ mice. To put this into perspective, it is useful to compare this reduction to that observed for agents that are well known to suppress CHS: UVB, platelet activating factor, and histamine suppress CHS responses by approximately 30-50% [46–48].

Our studies also show that anti-TNF-α neutralizing antibodies reverse the CHS defect observed in *Pparg*-/-^epi^ mice, but the antibodies had no effect on WT mice. Treatment with anti-TNF-α Abs, or loss of *Tnf* or TNFR-2, but not TNFR-1, results in a significant blockade of local UV-IS [18, 49–51]. However, the ability of anti-TNF-α neutralizing antibody treatment to suppress basal CHS responses is inconsistently seen in the literature [50, 52, 53]. Differences between studies on the basal CHS response in the presence of neutralizing anti-TNF-α antibodies could be due to the mouse genetic background, or possibly differences in the dosing, timing, and specificity of the antibody reagents.

Finally, the impaired CHS responses seen in *Pparg*-/-*epi* mice is reversed in *Pparg*-/-^epi^ mice crossed with *Tnf-/-* mice. As anti-TNF-α antibodies generally block both solTNF-α and tmTNF-α signaling [24], our studies are unable to determine whether solTNF-α or tmTNF-α signaling (or both) are necessary for the impaired CHS defect in *Pparg*-/-^epi^ mice. However, it is possible that increased tmTNF-α mediates the immunosuppression seen in *Pparg*-/-^epi^ mice: tmTNF-α is proposed to act through TNFR2 that is thought to be primarily involved in impaired CHS responses [21, 24, 25]. However, we cannot exclude the possibility that solTNF-α plays a key role as well since diffusion of this mediator may have reduced its presence within the epidermis. Future work is also necessary to determine whether epidermal TNF-α or TNF-α expressed by other cells is the primary determinant of the impaired CHS response in *Pparg*-/-^epi^ mice.

In addition to the defective CHS responses observed in *Pparg*-/-^epi^ mice, we also demonstrate that treatment with Rosig blocks UV-IS. UVB-induced immunosuppression is thought to play a key role in photocarcinogenesis and is associated with increased B16F10 melanoma tumor growth [9]. Our demonstration that Rosig treatment is able to completely block the ability of UVB treatments to promote B16F10 tumor growth suggests that this is due to the ability of Rosig to block UV-IS. However, it is also possible that increased inflammation in the tumor microenvironment may have also contributed to the increased tumor cell growth. In addition to the ability of Rosig treatment to block UV-induced tumor growth, we also show that B16F10 tumor cells grow faster in *Pparg*-/-^epi^ mice. This clearly demonstrates that epidermal PPARγ plays an important indirect role in B16F10 tumor growth. Since *Pparg*-/-^epi^ mice also have a profound defect in CHS responses, we speculate that increased tumor growth in *Pparg*-/-^epi^ mice is secondary to a defect in anti-tumor immune responses. However, additional work is necessary to further define the mechanisms and downstream effector cells that mediate the increased tumor growth in *Pparg*-/-^epi^ mice.

In conclusion, our current data indicates that epidermal PPARγ plays a robust role in modulating cutaneous inflammation, cutaneous immune responses and potentially anti-tumor immune surveillance. We also demonstrate that the ability of epidermal PPARγ to alter the photoresponse is largely dependent on TNF-α production. As a major organ exposed to environmental oxygen, the epidermis is constantly exposed to oxidative stress. Since endogenous PPARγ ligand production occurs non-enzymatically through lipid oxidation [54, 55], we therefore speculate that PPARγ may function to initiate a protective signaling pathway that serves to suppress inappropriately exuberant inflammatory responses and to promote robust immune system monitoring of oxidatively damaged cells within the epidermis or dermis.

## MATERIALS AND METHODS

### Reagents and chemicals

All chemicals were obtained from Sigma-Aldrich (St. Louis, MO, USA) unless indicated otherwise. Rosiglitazone maleate was obtained from Tecoland Corp (Irvine, CA, USA). Caspase-3/7 and caspase-8 activity assays were purchased from Promega (Caspase-Glo^®^ 8 and Caspase-Glo^®^ 3/7; Madison, WI, USA).

### Animals

The generation of wildtype (WT) and *Pparg*-/-^epi^ mice in the SKH1 background were previously described [5]. For mutation studies, Big Blue^®^ mice expressing the λLIZ shuttle vector in the C57Bl/6 background (Agilent Technologies, La Jolla, CA) were backcrossed with SKH-1 mice (Charles Rivers, Wilmington, MA) for 6 generations. These mice were then crossed with *Pparg*-/-^epi^ mice in the SKH-1 background to generate *Pparg*-/-^epi^ mice containing the λLIZ transgene. Wildtype (WT) sibling controls lacked the K14-Cre transgene. To generate *Pparg*-/-^epi^ mice with germline loss of *Tnf* (*Pparg*-/-^epi^ X *Tnf*-/-), B6.129S-*Tnf^tm1Gkl^*/J mice (Stock Number 005540, The Jackson Laboratory, Bar Harbor, ME) were backcrossed into the SKH-1 background for 6 generations prior to crossing with *Pparg*-/-^epi^ mice. For B16F10 tumor studies, B6.129-*Pparg*^tm2Rev^/J mice (Stock Number: 004584, The Jackson Laboratory) were crossed with B6N.Cg-Tg(KRT14-cre)1Amc/J mice (Stock Number 018964, The Jackson Laboratory) to generate epidermal-specific PPARγ knockout mice in the C57BL/6 background (C57.*Pparg*-/-^epi^ mice). Mice were housed under specific pathogen-free conditions at the Indiana University School of Medicine. The protocols were approved by the Indiana University School of Medicine Institutional Animal Care and Use Committee (IACUC).

### Acute UVB-induced apoptosis and inflammation

To assess the photoinflammatory and phototoxic responses, anesthetized mice were treated with 1500 J/m^2^ of UVB using FS40 lamps and the epidermis was removed after 24 hours for caspase 3 activity assessment as previously described [5]. Caspase 8 activity assays were performed essentially as described for the caspase 3 activity assays using Caspase-Glo^®^ reagents specific for caspase 8. To assess the acute inflammatory edema response, skin thickness was assessed as previously described [26].

### TNF-α qRT-PCR

Quantitative RT-PCR (qRT-PCR) was performed from RNA prepared from curetted epidermal scrapings of mouse skin using primers specific to murine *Tnf* transcript and 18S ribosomal RNA (RT^2^ qPCR Primer, Qiagen, Valencia, CA). Results were normalized using the 2^-ΔΔC^T method [56].

### TNF-α immunoblot

Mice were sacrificed by CO_2_ asphyxiation and a section of the dorsal skin was excised. The epidermis was isolated by thermolysin treatment using a modification of a protocol described by Ikehata *et al* [57]. Briefly, the skin was scraped to remove fat and muscle, and then was floated epidermal side up in 0.5ml/ml Thermolysin (sigma Alsdrich) in buffer (10 mM HEPES pH 7.4, 142mM NaCl, 6.7 mM KCl, 1 mM CaCl_2_), incubated at 37°C for 45 minutes. The epidermis was removed, snap frozen in liquid nitrogen, and stored at −80°C. Frozen skin samples were fragmented, suspended in modified RIPA buffer, (150 mM NaCl, 20 mM Tris-HCl pH 7.5, 1 mM EDTA, 1 mM EGTA, 1% Nonidet P40, 1% Sodium Deoxycholate, 0.1% SDS, 1 mM Na_3_VO_4_, 10 mM NaF, 0.2 mM Pefabloc, complete protease inhibitor (Roche Life Science). After further homogenization, the samples were then centrifuged and the supernatant was quantified for protein by BCA assay. 50 μg samples were run on SDS-PAGE and immunoblots performed with anti-TNF-α (clone D2D4, Cell Signaling Technology), followed by stripping and immunoblotting with anti-β-actin (clone AC-15, Sigma Aldrich).

### TNF-α immunohistochemistry

Immunolocalization of TNF-α protein expression in skin was performed on formalin-fixed paraffin-embedded sections. After deparaffinization, heat-induced antigen retrieval was performed in Borg Decloaker, RTU solution (Biocare Medical, Concord, CA, USA) in a pressure cooker for 20 minutes. Following non-specific protein and Fc-receptor blocking, immunohistochemical staining was performed using a polyclonal goat anti-TNF-α antibody (diluted 1:100; SC-1350, Santa Cruz Biotechnology, Dallas, TX), followed by donkey anti-goat-horseradish peroxidase secondary (1:200; SC-2020, Santa Cruz) and 3,3’-diaminobenzidine (DAB) chromagen detection.

### Contact hypersensitivity studies

Contact hypersensitivity studies utilized a modification of our previously described methodology [48]. Wildtype (WT), *Pparg*-/-^epi^, *Tnf*-/-, and double knockout (*Pparg*-/-^epi^ X*Tnf-*/- cross) in the SKH1 background were used for these studies. For rosiglitazone studies, mice were treated with rosiglitazone maleate (40 μg/ml) in water or regular water *ad libitum* for 10 days prior to UVB treatment and were maintained on oral rosiglitazone treatment throughout the experiments. The estimated daily intake was 8 mg/kg/day. Oral rosiglitazone has been shown to be systemically active in mice at doses of 3-8 mg/kg/day [58, 59]. An approximately 2.5 × 2.5-cm area of the distal back skin of these mice were treated with 7500 J/m^2^ UVB or were left untreated. After 5 days, mice were sensitized with topical 25 μl 0.5% DNFB in acetone:olive oil (4:1, v/v) on an area of the dorsal skin approximately 2.5 cm distant from the UVB-irradiated site. After 7 or 9 days, ear thickness was measured using a digital caliper and then 10 μl 0.5% DNFB was painted on the dorsal sides of one ear, whereas the other ear was painted with vehicle. After 24 hours, an elicitation reaction was measured by measuring the ear thickness as a final read out. Finally, post-DNFB ear thickness was subtracted from pre-DNFB ear thickness of the contralateral ear treated with vehicle alone (measured 24 hours earlier).

### Anti-TNF-α neutralization studies

WT and *Pparg-/-*^epi^ mice were injected intraperitoneally (ip) with 250 μg of either anti-TNF-α antibodies (clone XT3.11 *InVivo*MAb) or an isotype control antibody (rat IgG1) (both from Bio X Cell, West Lebanon, NH, USA). Injections were performed as indicated in the figure legends.

### B16F10 growth *in vitro*

B16F0 cells were maintained in RPMI 1640 (Mediatech) supplemented with 10% fetal bovine serum (Invitrogen) and 100 μg/ml penicillin and streptomycin (Invitrogen). B16F10 cells were plated onto a 96-well plate at 1250 cells/well. The next day, the media was replaced with media containing either vehicle (ethanol) or rosiglitazone maleate at concentrations ranging from 1-1000 nM. After 48 hours of growth, viable cells were quantitated by incubating the cells with 12 mM 3-(4,5-dimethylthiazol-2-yl)-2,5-diphenyltetrazolium bromide (MTT) per the manufacturer's instructions (Vybrant^®^ MTT Cell Proliferation Assay Kit, ThermoFisher Scientific, Waltham, MA, USA).

### B16F10 tumor growth *in vivo*

B16F10 tumor growth *in vivo* was performed in either C57BL/6 mice lacking epidermal *Pparg* (C57.*Pparg*-/-^epi^) or sibling controls lacking the K14-Cre allele (WT). Ten days prior to tumor cell implantation (day -10), treatment with either rosiglitazone or water was initiated as described above for the contact hypersensitivity studies. On day 0, the dorsal skin of C57BL/6 mice were shaved, and 5×10^5^ B16F10 cells were inoculated subcutaneously into the right flank of each mouse as described [9]. For UVB studies, mice were treated with 5000 J/m^2^ UVB on day 0, 6 and 12 on a 2.5 × 2.5 cm area of shaved dorsal skin which was approximately 3 cm away from the shielded tumor injection site. Tumor growth was measured daily with calipers as described [9]. When 5×10^5^ cells are used per injection, spontaneous tumor ulceration frequently occurs after 12 days. Invariably, this tumor ulceration is quickly followed by evidence of shock (unpublished observations). This required euthanasia per our approved protocol and limited our observation period for the UVB studies to 12 days. For this reason, when we examined B16F10 tumor cell growth in WT and C57.*Pparg*-/-^epi^ mice, we utilized a modification of this approach by implanting 10-fold less cells (5×10^4^). This allowed us to extend the period of time in which tumor growth was observed to 18 days.

### Cyclobutane pyrimidine dimer (CPD) clearance

WT and *Pparg*-/-^epi^ mice were treated with 1500 J/m^2^ UVB. At 1, 24 & 48 hrs, the mice were euthanized, the irradiated skin was removed and snap frozen, then the epidermis removed by scraping. After DNA isolation, the DNA was diluted to a concentration of 4 μg/ml in 0.1x PBS (pH7.4). For a standard curve, salmon sperm DNA (0.5 mg/ml in 0.1x PBS, pH7.4) was treated with increasing doses of UVB (0, 312.5, 625, 1250, 2500, or 5000 J/m^2^), then diluted to 4 μg/ml as above. Standard curve DNA and murine epidermal DNA were treated at 95°C for 10 min, rapidly placed on ice, and 50 μl of DNA or PBS alone was added to the wells of a protamine sulfate treated microwell plate. For protamine sulfate treatment, 0.003% protamine sulfate in H2O was prepared and 50 μl added to each well of 96-well plate. The plates were then allowed to dry overnight at 37°C prior to the addition of DNA. After the addition of denatured DNA, the samples were allowed to dry completely by incubating at 37°C. The plates were washed with PBS containing 0.05% Tween 20 (PBS-T), then blocked using CAS-Block™ (Life Technologies) diluted 1/25 in PBS containing 0.01 g/ml bovine serum albumin, and 0.5 μg/ml salmon sperm DNA. After incubating 30 min at 37°C, the plates were washed with PBS-T, and anti-thymine dimer HRP conjugate (Clone KTM53; Kamiya Biomedical, Seattle, WA, USA), diluted 1:2300 in PBS was added. After 1 hr at 37°C, the plates were washed and LumiGLO^®^ substrate (Cell Signaling Technology, Danvers, MA, USA) was added. Following luminescence detection, CPD levels in UV treated mouse epidermal DNA were then calculated as J/m^2^ equivalents based on the standard curve.

### Mutation frequency

Big Blue^®^ mice or related lacZ mice (MutaMouse) have been successfully used to study both UVA & UVB-induced mutagenesis [60, 61]. For mutation studies, WT and *Pparg-/-*^epi^ mice expressing the λLIZ transgene were anesthetized and treated with increasing doses of UVB. Control mice were not UVB treated. Five days later we euthanized the mice and excised the treated skin for epidermal DNA isolation. We waited 5 days before euthanasia to allow for both complete repair of CPD lesions and for clearance of mutations in non-cancer causing suprabasal cells through epidermal turnover (epidermal transit time = 3-5 days for hairless mouse epidermis) [62, 63]. For DNA isolation, we separated the epidermis from the subcutaneous tissue using thermolysin as described above. Cells of the epidermis were isolated by trypsinization in 0.25% trypsin and the cellular nuclei DNA was isolated using the RecoverEase^™^ DNA Isolation Kit (Agilent Technologies). Samples were dialyzed in TE buffer using G Biosciences Tube-O-Dialyzer. The mutation frequency was determined using Agilent Transpack Packaging Extract and λSelect-cII Mutation Detection System for Big Blue Rodents.

### Statistical analysis

Group comparisons are shown as mean and standard error and were analyzed for statistical significance as detailed in each figure legend using Graphpad Prism 5.0 (Graphpad Software, Inc., San Diego, CA, USA).

## SUPPLEMENTARY MATERIALS FIGURES AND TABLES



## Abbreviations

CHS: Contact hypersensitivity
CPD: Cyclobutane pyrimidine dimer
DNA: Deoxyribonucleic acid
DNFB: 2,4-Dinitrofluorobenzene
EIA: Enzyme immunoassay
IL: Interleukin
MF: Mutation frequency
NER: Nucleotide excision repair
PPAR: Peroxisome proliferator-activated receptor
PPRE: Peroxisome proliferator response element
RT-PCR: Reverse transcription-polymerase chain reaction
RXR: Retinoid X receptor
SEM: Standard error of the mean
TNF: Tumor necrosis factor
solTNF-α: soluble TNF-α
tmTNF-α: transmembrane TNF-α
Th: T helper
UV: Ultraviolet
UV-IS: Ultraviolet-induced immunosuppression
WT: Wildtype

## References

[1] Konger RL, Marathe GK, Yao Y, Zhang Q, Travers JB (2008). Oxidized glycerophosphocholines as biologically active mediators for ultraviolet radiation-mediated effects. Prostaglandins Other Lipid Mediat.

[2] Ricote M, Glass CK (2007). PPARs and molecular mechanisms of transrepression. BBA-Mol Cell Bio L.

[3] Hatton JL, Yee LD (2008). Clinical Use of PPARγ Ligands in Cancer. PPAR Research.

[4] Robbins GT, Nie D (2012). PPAR gamma, bioactive lipids, and cancer progression. Front Biosci (Landmark Ed).

[5] Sahu RP, DaSilva SC, Rashid B, Martel KC, Jernigan D, Mehta SR, Mohamed DR, Rezania S, Bradish JR, Armstrong AB, Warren S, Konger RL (2012). Mice lacking epidermal PPARγ exhibit a marked augmentation in photocarcinogenesis associated with increased UVB-induced apoptosis, inflammation and barrier dysfunction. Int J Cancer.

[6] Indra AK, Castaneda E, Antal MC, Jiang M, Messaddeq N, Meng X, Loehr CV, Gariglio P, Kato S, Wahli W, Desvergne B, Metzger D, Chambon P (2007). Malignant transformation of DMBA/TPA-induced papillomas and nevi in the skin of mice selectively lacking retinoid-X-receptor alpha in epidermal keratinocytes. J Invest Dermatol.

[7] Nicol CJ, Yoon M, Ward JM, Yamashita M, Fukamachi K, Peters JM, Gonzalez FJ (2004). PPARgamma influences susceptibility to DMBA-induced mammary, ovarian and skin carcinogenesis. Carcinogenesis.

[8] Kripke ML (2013). Reflections on the field of photoimmunology. J Invest Dermatol.

[9] Sahu RP, Turner MJ, DaSilva SC, Rashid BM, Ocana JA, Perkins SM, Konger RL, Touloukian CE, Kaplan MH, Travers JB (2012). The environmental stressor ultraviolet B radiation inhibits murine antitumor immunity through its ability to generate platelet-activating factor agonists. Carcinogenesis.

[10] Yang XY, Wang LH, Farrar WL (2008). A Role for PPARγ in the Regulation of Cytokines in Immune Cells and Cancer. PPAR Research.

[11] Dahten A, Koch C, Ernst D, Schnöller C, Hartmann S, Worm M (2008). Systemic PPARgamma ligation inhibits allergic immune response in the skin. J Invest Dermatol.

[12] Behshad R, Cooper KD, Korman NJ (2008). A retrospective case series review of the peroxisome proliferator-activated receptor ligand rosiglitazone in the treatment of atopic dermatitis. Arch Dermatol.

[13] Wu L, Wang G, Qu P, Yan C, Du H (2011). Overexpression of dominant negative peroxisome proliferator-activated receptor-γ (PPARγ) in alveolar type II epithelial cells causes inflammation and T-cell suppression in the lung. Am J Pathol.

[14] You YH, Lee DH, Yoon JH, Nakajima S, Yasui A, Pfeifer GP (2001). Cyclobutane pyrimidine dimers are responsible for the vast majority of mutations induced by UVB irradiation in mammalian cells. J Biol Chem.

[15] Briganti S, Flori E, Bellei B, Picardo M (2014). Modulation of PPARγ provides new insights in a stress induced premature senescence model. PLoS One.

[16] Bruemmer D, Yin F, Liu J, Berger JP, Sakai T, Blaschke F, Fleck E, Van Herle AJ, Forman BM, Law RE (2003). Regulation of the growth arrest and DNA damage-inducible gene 45 (GADD45) by peroxisome proliferator-activated receptor gamma in vascular smooth muscle cells. Circ Res.

[17] Clydesdale GJ, Dandie GW, Muller HK (2001). Ultraviolet light induced injury: immunological and inflammatory effects. Immunol Cell Biol.

[18] Hart PH, Grimbaldeston MA, Swift GJ, Sedgwick JD, Körner H, Finlay-Jones JJ (1998). TNF modulates susceptibility to UVB-induced systemic immunomodulation in mice by effects on dermal mast cell prevalence. Eur J Immunol.

[19] Arnott CH, Scott KA, Moore RJ, Robinson SC, Thompson RG, Balkwill FR (2004). Expression of both TNF-alpha receptor subtypes is essential for optimal skin tumour development. Oncogene.

[20] Moore RJ, Owens DM, Stamp G, Arnott C, Burke F, East N, Holdsworth H, Turner L, Rollins B, Pasparakis M, Kollias G, Balkwill F (1999). Mice deficient in tumor necrosis factor-α are resistant to skin carcinogenesis. Nat Med.

[21] Wang B, Fujisawa H, Zhuang L, Kondo S, Shivji GM, Kim CS, Mak TW, Sauder DN (1997). Depressed Langerhans cell migration and reduced contact hypersensitivity response in mice lacking TNF receptor p75. J Immunol.

[22] Zhao X, Rong L, Zhao X, Li X, Liu X, Deng J, Wu H, Xu X, Erben U, Wu P, Syrbe U, Sieper J, Qin Z (2012). TNF signaling drives myeloid-derived suppressor cell accumulation. J Clin Invest.

[23] Olleros ML, Vesin D, Lambou AF, Janssens JP, Ryffel B, Rose S, Frémond C, Quesniaux VF, Szymkowski DE, Garcia I (2009). Dominant-negative tumor necrosis factor protects from Mycobacterium bovis Bacillus Calmette Guérin (BCG) and endotoxin-induced liver injury without compromising host immunity to BCG and Mycobacterium tuberculosis. J Infect Dis.

[24] Horiuchi T, Mitoma H, Harashima S, Tsukamoto H, Shimoda T (2010). Transmembrane TNF-α: structure, function and interaction with anti-TNF agents. Rheumatology (Oxford).

[25] Hu X, Li B, Li X, Zhao X, Wan L, Lin G, Yu M, Wang J, Jiang X, Feng W, Qin Z, Yin B, Li Z (2014). Transmembrane TNF-α promotes suppressive activities of myeloid-derived suppressor cells via TNFR2. J Immunol.

[26] Konger RL, Derr-Yellin E, Hojati D, Lutz C, Sundberg JP (2016). Comparison of the acute ultraviolet photoresponse in congenic albino hairless C57BL/6J mice relative to outbred SKH1 hairless mice. Exp Dermatol.

[27] Tsuru K, Horikawa T, Budiyanto A, Hikita I, Ueda M, Ichihashi M (2001). Low-dose ultraviolet B radiation synergizes with TNF-alpha to induce apoptosis of keratinocytes. J Dermatol Sci.

[28] Fulda S, Debatin KM (2006). Extrinsic versus intrinsic apoptosis pathways in anticancer chemotherapy. Oncogene.

[29] Wang Z, Coleman DJ, Bajaj G, Liang X, Ganguli-Indra G, Indra AK (2011). RXR[alpha] Ablation in Epidermal Keratinocytes Enhances UVR-Induced DNA Damage, Apoptosis, and Proliferation of Keratinocytes and Melanocytes. J Invest Dermatol.

[30] Stallons LJ, McGregor WG (2010). Translesion Synthesis Polymerases in the Prevention and Promotion of Carcinogenesis. J Nucleic Acids.

[31] D'Errico M, Lemma T, Calcagnile A, Proietti De Santis L, Dogliotti E (2007). Cell type and DNA damage specific response of human skin cells to environmental agents. Mutat Res.

[32] Harbach PR, Zimmer DM, Filipunas AL, Mattes WB, Aaron CS (1999). Spontaneous mutation spectrum at the lambda cII locus in liver, lung, and spleen tissue of Big Blue transgenic mice. Environ Mol Mutagen.

[33] Chen JH, Chang JL, Chen PR, Chuang YJ, Tang ST, Pan SF, Lin TB, Chen KH, Chen MJ (2014). Inhibition of Peroxisome Proliferator-Activated Receptor Gamma Prevents the Melanogenesis in Murine B16/F10 Melanoma Cells. BioMed Res Int.

[34] Liu K, Black RM, Acton JJ, Mosley R, Debenham S, Abola R, Yang M, Tschirret-Guth R, Colwell L, Liu C, Wu M, Wang CF, MacNaul KL (2005). Selective PPARgamma modulators with improved pharmacological profiles. Bioorg Med Chem Lett.

[35] Askling J, Fahrbach K, Nordstrom B, Ross S, Schmid CH, Symmons D (2011). Cancer risk with tumor necrosis factor alpha (TNF) inhibitors: meta-analysis of randomized controlled trials of adalimumab, etanercept, and infliximab using patient level data. Pharmacoepidemiol Drug Saf.

[36] Kimball AB, Schenfeld J, Accortt NA, Anthony MS, Rothman KJ, Pariser D (2014). Incidence rates of malignancies and hospitalized infectious events in patients with psoriasis with or without treatment and a general population in the U.S.A.: 2005-09. Br J Dermatol.

[37] Moulis G, Sommet A, Béné J, Montastruc F, Sailler L, Montastruc JL, Lapeyre-Mestre M (2012). Cancer risk of anti-TNF-α at recommended doses in adult rheumatoid arthritis: a meta-analysis with intention to treat and per protocol analyses. PLoS One.

[38] Vink AA, Shreedhar V, Roza L, Krutmann J, Kripke ML (1998). Cellular target of UVB-induced DNA damage resulting in local suppression of contact hypersensitivity. J Photochem Photobiol B.

[39] Zhang W, Hanks AN, Boucher K, Florell SR, Allen SM, Alexander A, Brash DE, Grossman D (2005). UVB-induced apoptosis drives clonal expansion during skin tumor development. Carcinogenesis.

[40] Chaturvedi V, Qin JZ, Denning MF, Choubey D, Diaz MO, Nickoloff BJ (1999). Apoptosis in proliferating, senescent, and immortalized keratinocytes. J Biol Chem.

[41] da Rocha LF, Dantas AT, Duarte AL, de Melo Rego MJ, Pitta Ida R, Pitta MG (2013). Agonists in Adaptive Immunity: What Do Immune Disorders and Their Models Have to Tell Us?. PPAR Res.

[42] Klotz L, Dani I, Edenhofer F, Nolden L, Evert B, Paul B, Kolanus W, Klockgether T, Knolle P, Diehl L (2007). Peroxisome proliferator-activated receptor gamma control of dendritic cell function contributes to development of CD4+ T cell anergy. J Immunol.

[43] Wu L, Yan C, Czader M, Foreman O, Blum JS, Kapur R, Du H (2012). Inhibition of peroxisome proliferator-activated receptor-γ in myeloid lineage cells induces systemic inflammation, immunosuppression and tumorigenesis. Blood.

[44] Tachibana M, Wada K, Katayama K, Kamisaki Y, Maeyama K, Kadowaki T, Blumberg RS, Nakajima A (2008). Activation of peroxisome proliferator-activated receptor gamma suppresses mast cell maturation involved in allergic diseases. Allergy.

[45] Adapala N, Chan MM (2008). Long-term use of an antiinflammatory, curcumin, suppressed type 1 immunity and exacerbated visceral leishmaniasis in a chronic experimental model. Laboratory Investigation.

[46] Ferracini M, Sahu RP, Harrison KA, Waeiss RA, Murphy RC, Jancar S, Konger RL, Travers JB (2015). Topical photodynamic therapy induces systemic immunosuppression via generation of platelet-activating factor receptor ligands. J Invest Dermatol.

[47] Sahu RP, Petrache I, Van Demark MJ, Rashid BM, Ocana JA, Tang Y, Yi Q, Turner MJ, Konger RL, Travers JB (2013). Cigarette smoke exposure inhibits contact hypersensitivity via the generation of platelet-activating factor agonists. J Immunol.

[48] Zhang Q, Yao Y, Konger RL, Sinn AL, Cai S, Pollok KE, Travers JB (2008). UVB radiation-mediated inhibition of contact hypersensitivity reactions is dependent on the platelet-activating factor system. J Invest Dermatol.

[49] Amerio P, Toto P, Feliciani C, Suzuki H, Shivji G, Wang B, Sauder DN (2001). Rethinking the role of tumour necrosis factor-α in ultraviolet (UV) B-induced immunosuppression: altered immune response in UV-irradiated TNFR1R2 gene-targeted mutant mice. Br J Dermatol.

[50] Bromberg JS, Chavin KD, Kunkel SL (1992). Anti-tumor necrosis factor antibodies suppress cell-mediated immunity in vivo. J Immunol.

[51] Chai OH, Lee HK, Lee YC, Lee MS, Han EH, Kim HT, Song CH (2005). Roles of TNF-alpha and IgE in the late phase of contact hypersensitivity induced by trimellitic anhydride. Exp Mol Med.

[52] Cumberbatch M, Kimber I (1995). Tumour necrosis factor-alpha is required for accumulation of dendritic cells in draining lymph nodes and for optimal contact sensitization. Immunology.

[53] Moodycliffe AM, Kimber I, Norval M (1994). Role of tumour necrosis factor-alpha in ultraviolet B light-induced dendritic cell migration and suppression of contact hypersensitivity. Immunology.

[54] Zhang Q, Seltmann H, Zouboulis CC, Konger RL (2006). Involvement of PPARgamma in oxidative stress-mediated prostaglandin E(2) production in SZ95 human sebaceous gland cells. J Invest Dermatol.

[55] Zhang Q, Southall MD, Mezsick SM, Johnson C, Murphy RC, Konger RL, Travers JB (2005). Epidermal peroxisome proliferator-activated receptor γ as a target for ultraviolet B radiation. J Biol Chem.

[56] Livak KJ, Schmittgen TD (2001). Analysis of relative gene expression data using real-time quantitative PCR and the 2(-μ μ. C)(T) Method. Methods.

[57] Ikehata H, Aiba S, Ozawa H, Ono T (2001). Thermolysin improves mutation analysis in skin epidermis from ultraviolet light-irradiated Muta Mouse. Environ Mol Mutagen.

[58] Hwang J, Kleinhenz DJ, Rupnow HL, Campbell AG, Thulé PM, Sutliff RL, Hart CM (2007). The PPARgamma ligand, rosiglitazone, reduces vascular oxidative stress and NADPH oxidase expression in diabetic mice. Vascul Pharmacol.

[59] Hindlet P, Barraud C, Boschat L, Farinotti R, Bado A, Buyse M (2012). Rosiglitazone and metformin have opposite effects on intestinal absorption of oligopeptides via the proton-dependent PepT1 transporter. Mol Pharmacol.

[60] Ikehata H, Kudo H, Masuda T, Ono T (2003). UVA induces C->T transitions at methyl-CpG-associated dipyrimidine sites in mouse skin epidermis more frequently than UVB. Mutagenesis.

[61] Frijhoff AF, Rebel H, Mientjes EJ, Kelders MC, Steenwinkel MJ, Baan RA, van Zeeland AA, Roza L (1997). UVB-induced mutagenesis in hairless λ lacZ-transgenic mice. Environ Mol Mutagen.

[62] Thorud E, Clausen OP, Aarnaes E (1988). Turnover and maturation kinetics in the hairless mouse epidermis. Continuous [3H]TdR labelling and mathematical model analyses. Cell Tissue Kinet.

[63] Potten CS (1975). Epidermal cell production rates. J Invest Dermatol.

