# Supplementary material for: Epidermal PPARγ influences subcutaneous tumor growth and acts through TNF-α to regulate contact hypersensitivity and the acute photoresponse
Source: Oncotarget. 2017 Sep 18;8(58):98184–99. doi: 10.18632/oncotarget.21002 (PMC5716722; doi:10.18632/oncotarget.21002)
Supplement: Supplementary file 1 [file oncotarget-08-98184-s001.pdf]

Epidermal PPARγ influences subcutaneous tumor growth and acts through TNF-α to regulate contact hypersensitivity and the acute photoresponse

SUPPLEMENTARY MATERIALS

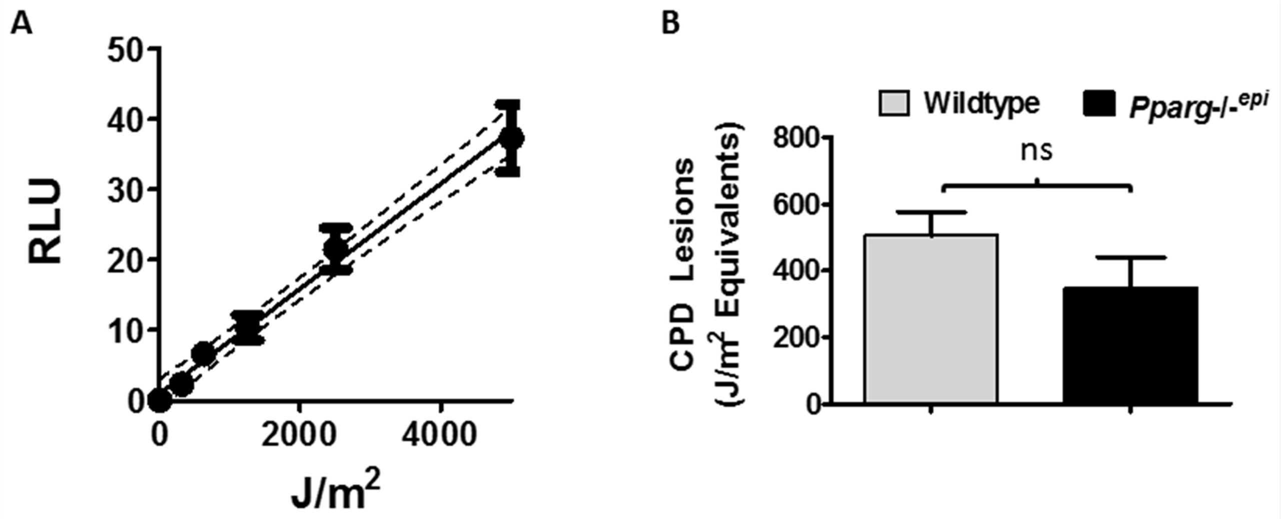

Supplementary Figure 1: Standard curve and initial CPD lesion levels in wildtype and *Pparg*-/-^{epi} mice following UVB. (A). *Typical standard curve assay* for CPD assay. Salmon sperm DNA (0.5 mg/ml) was treated with increasing doses of UVB (0, 312.5, 625, 1250, 2500, or 5000j/m^2), then diluted to 4µg/ml for enzyme immunoassay using an anti-thymine dimer horseradish peroxidase conjugate. Relative light units (RLU) obtained following the addition of LumoGLO® substrate were then plotted against J/m^2 equivalents to obtain CPD levels (J/m^2 equivalents) for unknown samples. **(B)**. Initial CPD lesion quantity obtained in wild type and *Pparg*-/-^{epi} at 1 hour after UVB irradiation. Mice were treated with a single 1500j/m^2 dose of UVB. results are as show the mean and SEM of the CPD lesion calculated as a J/m^2 equivalents based on the standard curve. p=0.1971, 2-tailed *t-test*; n=5 *Pparg*-/-^{epi} and 7 WT mice.
